# Impact of Anchoring Groups on the Photocatalytic Performance of Iridium(III) Complexes and Their Toxicological Analysis

**DOI:** 10.3390/molecules29112564

**Published:** 2024-05-30

**Authors:** Xiao Yao, Linyu Fan, Qian Zhang, Chaoqun Zheng, Xue Yang, Yisang Lu, Yachen Jiang

**Affiliations:** 1 School of Ecological Environment and Urban Construction, Fujian University of Technology, Fuzhou 350118, China; 2 Department of Applied Biology and Chemical Technology, The Hong Kong Polytechnic University, Hung Hom, Hong Kong, China; 3 PolyU Shenzhen Research Institute, Shenzhen 518057, China

**Keywords:** Ir(III) cyclometalated, anchoring ligand, carbazole, photosensitizers, biquinoline

## Abstract

Three different iridium(III) complexes, labelled as **Ir1**–**Ir3**, each bearing a unique anchoring moiety (diethyl [2,2′-bipyridine]-4,4′-dicarboxylate, tetraethyl [2,2′-bipyridine]-4,4′-diylbis(phosphonate), or [2,2′-biquinoline]-4,4′-dicarboxylic acid), were synthesized to serve as photosensitizers. Their electrochemical and photophysical characteristics were systematically investigated. ERP measurements were employed to elucidate the impact of the anchoring groups on the photocatalytic hydrogen generation performance of the complexes. The novel iridium(III) complexes were integrated with platinized TiO_2_ (Pt–TiO_2_) nanoparticles and tested for their ability to catalyze hydrogen production under visible light. A H_2_ turnover number (TON) of up to 3670 was obtained upon irradiation for 120 h. The complexes with tetraethyl [2,2′-bipyridine]-4,4′-diylbis(phosphonate) anchoring groups were found to outperform those bearing other moieties, which may be one of the important steps in the development of high-efficiency iridium(III) photosensitizers for hydrogen generation by water splitting. Additionally, toxicological analyses found no significant difference in the toxicity to luminescent bacteria of any of the present iridium(III) complexes compared with that of TiO_2_, which implies that the complexes investigated in this study do not pose a high risk to the aquatic environment compared to TiO_2_.

## 1. Introduction

In light of the increasing demand for energy and diminishing global fossil fuel supply, the quest for alternative energy sources has evolved into a critical and intricate field of research [1]. Solar power surfaced as a viable solution to contemporary energy challenges owing to its sustainability and zero-carbon footprint [2]. Photocatalytic technology enables the effective harnessing and conversion of this energy type into various forms of power [3,4,5,6,7,8,9,10]. Particularly, the solar-driven photocatalytic water splitting technique offers an easy procedure for producing hydrogen fuel [11,12], a concept initially introduced in 1972 by Fujishima and Honda by utilising a TiO_2_ photoanode [13]. After this study, the technology for hydrogen production through water splitting has evolved significantly [14,15].

Iridium(III) cyclometalated complexes, distinguished for their role in dye fabrication, have significantly contributed to the field of photocatalytic hydrogen production [16]. These complexes are essential for their superior ligand-field stabilisation energy, primarily attributed to the 5 d valence shell, especially when compared with other metal dyes derived from first- and second-row transition metals [2,17]. Research led by Bernhard et al. [2,18,19,20] has explored the potential of iridium(III) complexes, particularly those following the formula [Ir(C^N)_2_(N^N)]⁺ (where C^N represents the cyclometalating ligand and N^N denotes the anchoring group), in applications related to photochemical water splitting for hydrogen production. Impressive results, such as a turnover number (TON) of 800, were achieved utilizing 50 μM of [Ir(ppy)_2_(bpy)]⁺ as a photosensitizer within a water–acetonitrile solvent mixture at a ratio of 1:1 (*v*/*v*) [18,21,22,23,24]. Subsequently, Ir(III) cyclometalated complexes have garnered interest for their potential applications as photosensitizers [25,26,27]. The stability of these complexes is enhanced through C^N ligands, which provide σ-donation from carbon atoms bonded to the metal, increasing electron density at the metal core and improving resilience during photocatalytic experiments [2,28]. Furthermore, physicochemical properties, including energy gaps, can be effectively tuned by varying the ligands [29,30], making Ir(III) cyclometalated complexes attractive candidates for dyes in H_2_ production through water splitting.

However, Ir(III) cyclometalated complexes often demonstrate weak visible light absorption, constituting approximately 40% of the sunlight spectrum [26,30,31,32,33,34]. Adjustments to the structures of Ir(III) cyclometalated complexes are necessary to overcome these limitations and enhance their hydrogen output. The potential for enhancing hydrogen evolution performance in iridium-based photosensitizers remains unexplored. Despite their potential, developing efficient and stable Ir(III) photosensitizers remains crucial for improving the kinetics of hydrogen production. Recent advancements in the field of Ir(III) photosensitizers focus on altering the structure of cyclometalating and ancillary ligands to modify their photochemical and physical attributes [3,16,35,36]. Strategic choices in chromophores and molecular structures can enhance light absorption, electron transfer efficiency, and photosensitizer longevity [37]. Optimizing the donor groups on cyclometalating ligands in Ir(III) photosensitizers enhances spectral responses, accelerates charge transfer, increases stability, and ensures better energy level alignment with TiO_2_ conduction band edges, thereby boosting photocatalytic hydrogen production efficacy [38]. Carbazole (Cz), especially 9-phenyl-9H-carbazole, has been widely used in the synthesis of conjugated microporous polymers for photocatalysis owing to its excellent electron-donating and charge-transporting properties and its suitability for post-functionalization [39,40]. The isoquinoline functional groups have also found extensive application in dyes for water-splitting reactions owing to their significant charge transfer ability [41]. Combining Cz with furan and isoquinoline to form a donor–π–acceptor (D–π–A) framework enhances strong intramolecular charge transfer (ICT) capabilities, resulting in high molar extinction coefficients in the resultant iridium(III) complexes [42,43].

Previous studies on Ir(III) cyclometalated complexes have found that different anchoring groups on N^N ligands are used to achieve highly efficient and stable water-splitting systems [44,45]. Incorporating anchoring groups, including carboxylate, or phosphonic acid, into the bipyridine ligand structure of [Ir(C^N)_2_(N^N)]⁺-type dyes [45] facilitates their stable attachment to semiconductors. The presence of a 2,2′-bipyridine or biquinoline moiety in these anchoring groups significantly enhances the molar extinction coefficients of these complexes, thereby increasing their light absorption capacity, which is crucial for photocatalytic water splitting to generate hydrogen [46]. Complexes with carboxyl functional groups on their anchoring ligands establish strong connections with semiconductors such as TiO_2_. This results in improved electron transport and hydrogen production and increased water solubility due to the acid–base equilibrium in Ir(III) cyclometalated complexes [47,48,49,50]. However, the stability of these dyes under photocatalytic conditions may pose a challenge [51], with the hydrolysis of carboxylate linkages identified as a limiting factor in the efficiency of electron transfer from the photosensitizer to the TiO_2_ surface [52]. In contrast, phosphonate linkages demonstrate enhanced stability when bonded to TiO_2_ surfaces, surpassing the performance of carboxylate linkages [26,52].

**Figure 1:**
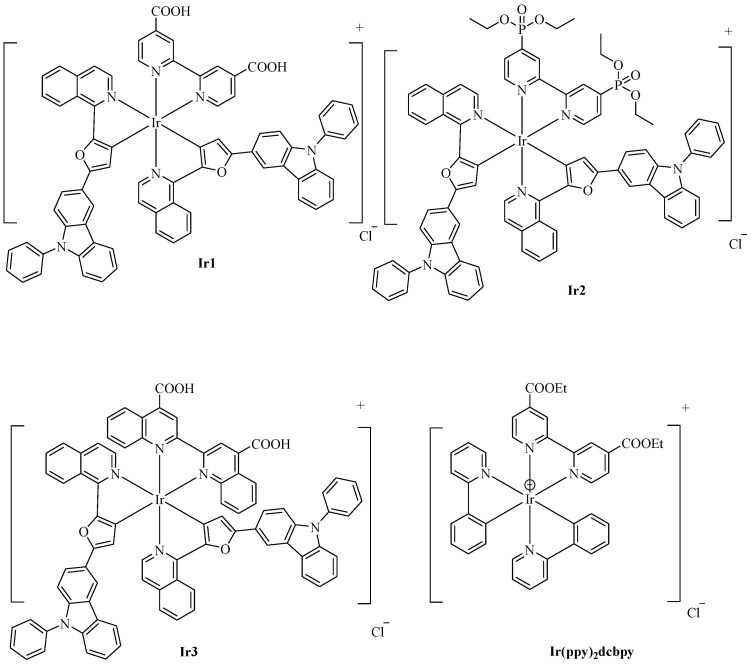
Chemical structures of iridium(III) dyes **Ir1**–**Ir3** and **[Ir(ppy)_2_(dcbpy)]Cl**.

Three Ir(III) complexes have been synthesized, denoted as **Ir1** to **Ir3**. These complexes feature diethyl [2,2′-bipyridine]-4,4′-dicarboxylate, tetraethyl [2,2′-bipyridine]-4,4′-diylbis(phosphonate), or [2,2′-Biquinoline]-4,4′-dicarboxylic acid as anchoring units. They are designed to serve as photosensitizers in light-driven hydrogen production through water splitting (Figure 1). The dye molecular structure includes 3-(5-(isoquinolin-1-yl)furan-2-yl)-9-phenyl-9H-carbazole motifs, forming a donor–π–acceptor (D–π–A) architecture [42,43,53,54,55,56]. Furthermore, [Ir(ppy)_2_(dcbpy)]Cl was synthesized to serve as a benchmark for evaluating the light-harvesting abilities and hydrogen generation efficiency of these synthesized dyes. Therefore, this study aims to investigate the effect of the C^N ligands and anchoring units on the properties of these complexes through a comprehensive analysis employing both electrochemical and photophysical techniques. Furthermore, systematic investigations into their photocatalytic hydrogen production capabilities through water splitting were undertaken, including exploring the correlation between the anchoring groups and their hydrogen generation activities. Additionally, toxicology research was conducted to check their environmental influence.

## 2. Results and Discussion

### 2.1. Synthesis and Characterization

**Scheme 1:**
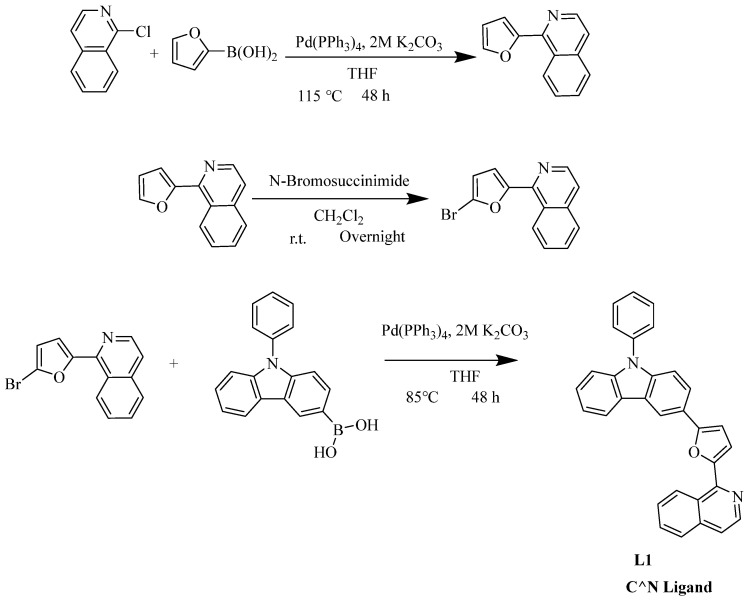
Synthetic route for cyclometalating ligand.

**Scheme 2:**
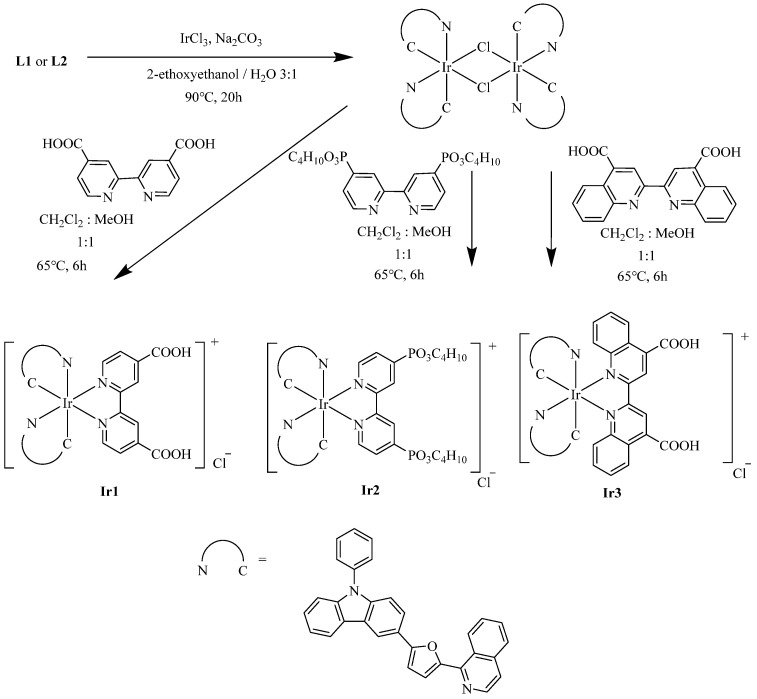
Synthetic route for iridium(III) complexes **Ir1**–**Ir3**.

Scheme 1 and Scheme 2 illustrate the synthetic methodologies for the C^N ligands and Ir(III) photosensitizers, respectively. Photosensitizers structure consists of an Ir(III) core surrounded by two C^N cyclometalating ligands and a variable N^N auxiliary ligand, which is diethyl [2,2′-bipyridine]-4,4′-dicarboxylate, tetraethyl [2,2′-bipyridine]-4,4′-diylbis(phosphonate), or [2,2′-Biquinoline]-4,4′-dicarboxylic acid. The C^N ligand was synthesized utilizing the Suzuki coupling reaction. For Ir(III) complexes (**Ir1**–**Ir3**) generation, a two-stage synthetic strategy was employed. Initially, μ-chloride-bridged dimeric complexes were synthesized by reacting Ir(III) chloride hydrate with the C^N cyclometalating ligand [57,58]. These dimers were subsequently reacted with the N^N auxiliary ligands to form the final Ir(III) complexes (**Ir1**–**Ir3**). These Ir(III) complexes demonstrate stability as solid substances under atmospheric conditions.

All synthesized organic precursors and ligands underwent characterization using ^1^H and ^13^C NMR spectroscopic techniques. Additionally, a comprehensive analysis of the Ir(III) complexes was performed utilizing a combination of LC-ESI-Q-TOF MS and 1H NMR spectroscopy. A distinct peak observed between approximately 8.74 to 9.28 ppm in the ^1^H NMR spectra is attributed to the ortho proton of isoquinoline [11]. These spectroscopic findings corroborate the anticipated molecular structures of the complexes. However, obtaining high-quality ^13^C NMR spectra proved challenging owing to the complex limited solubility in commonly used organic solvents.

### 2.2. Photophysical Properties of Iridium(III) Dyes

**Figure 2:**
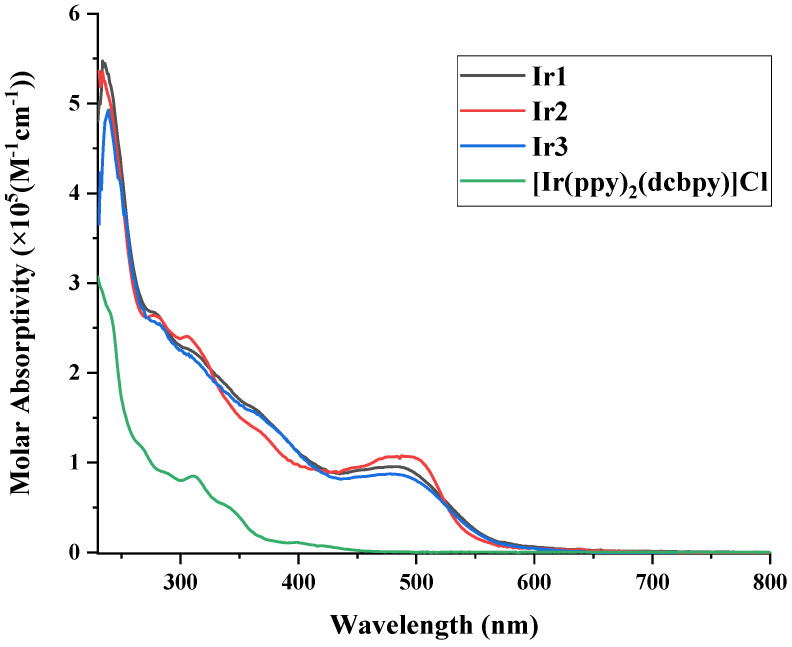
UV–Vis absorption spectra of **Ir1** to **Ir3** and **[Ir(ppy)_2_(dcbpy)]Cl** in CH_2_Cl_2_ at 293 K.

The photophysical characteristics of Ir(III) complexes were investigated in dichloromethane at ambient temperature (293 K). Table 1 lists their unique UV/Vis absorption spectra, while Figure 2 graphically represents them. **Ir1** to **Ir3** demonstrates significant absorption of approximately 230 nm in the UV region, which is attributed to intra-ligand charge transfer transitions [59]. The prominent band observed at approximately 400 nm is owing to spin-allowed π to π* electronic transitions within the C^N and N^N ligands [36]. Both metal-to-ligand charge transfer (MLCT) and ligand-to-ligand charge transfer (LLCT) contribute to the extensive absorption bands observed around 400 nm [60]. These compounds demonstrated superior absorption intensity in the visible spectrum than in the conventional **[Ir(ppy)_2_(dcbpy)]Cl** complex, indicating enhanced light-harvesting efficiency in **Ir1**–**Ir3** over **[Ir(ppy)_2_(dcbpy)]Cl** [30,61,62]. The broadened absorption spectra with higher ε values in 500 nm range result from the extended π-conjugation provided by the electron-donating Cz group in the Ir(III) dyes [63]. In addition, **Ir3** showed a slight redshift with lower molar absorptivity compared to **Ir1**, possibly owing to the extension of conjugation in the anchoring group [39].

**Figure 3:**
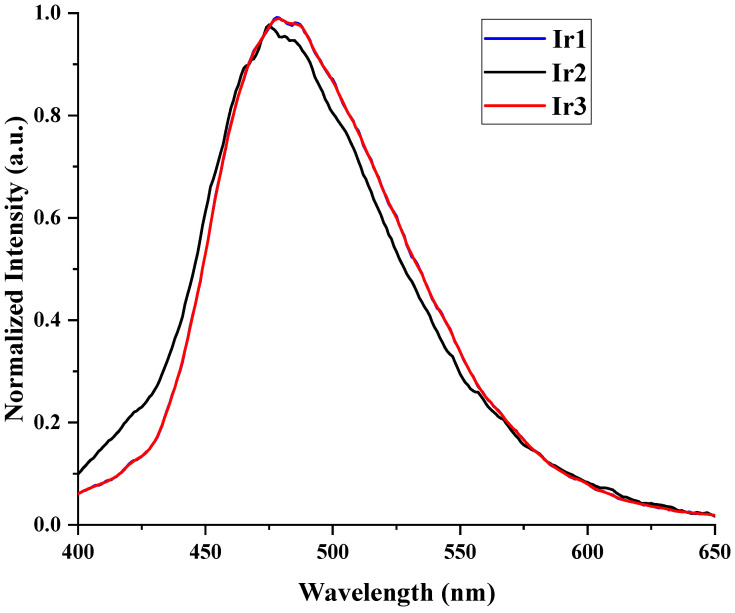
PL spectra of **Ir1**–**Ir3** in dichloromethane solution at 293 K.

Figure 3 presents the photoluminescence profiles of **Ir1** to **Ir3** in a dichloromethane solution at 293 K. Upon photochemical excitation at 480 nm, distinct emission spectra are observed for all Ir(III) dyes. These emissions originate from a combination of ^3^MLCT and LC ^3^π to π* transitions [64,65,66]. The photoluminescence spectra of **Ir1** and **Ir3** demonstrate only negligible change, indicating the same energy levels. This observation is consistent with the electrochemical data.

### 2.3. Electrochemical Properties of Iridium(III) Dyes

Optimizing the energy gap between the TiO_2_ semiconductor and sacrificial electron donor in Ir(III) complexes is crucial for efficient hydrogen production through water splitting. Electron transfer efficacy and charge separation in these complexes depends on the LUMO energy level being higher than the conduction band of the semiconductor and the HOMO level being below the sacrificial electron donor. To elucidate the energy profiles of the Ir(III) complexes, cyclic voltammetry (CV) was employed utilizing a standard three-electrode set-up. Table 2 summarizes the comprehensive parameters derived from these experiments.

The CV results demonstrated significant differences compared to those obtained for **[Ir(ppy)_2_(dcbpy)]Cl**, indicating that the different ligands significantly influenced the energy gaps in Ir(III) dyes [29,30]. The CV data indicate that the conduction band of the TiO_2_ semiconductor, at −4.4 eV, was lower than the E_LUMO_ levels of all Ir(III) dyes, ranging from −3.60 to −3.49 eV. This difference facilitates effective electron injection during light-induced hydrogen production [67]. The *E*_HOMO_ values of **Ir1**–**Ir3** were −5.66 or −5.65 V, which was more negative than the redox potentials of the sacrificial electron donor (AA) (−4.65 eV, pH~4) [68,69], thereby enabling effective dye regeneration from the sacrificial electron donor. All the Ir(III) dyes demonstrated energy levels that meet the prerequisites for efficient electron injection and charge separation, highlighting their potential suitability for hydrogen production through water splitting.

Previous research indicates that the HOMO energy levels are primarily located at the Ir centre and on cyclometalating C^N ligands and can be readily adjusted. In contrast, the LUMO energy levels are distributed across the anchoring N^N groups [26,57]. The phosphonate anchoring groups establish a stronger chemical linkage to the TiO_2_ surface, thereby tuning the LUMO energy level and enhancing the water-splitting hydrogen generation [45]. The energy level of **Ir1** and **Ir3** are quite the same, as predicted by PL data previously (Figure 3).

### 2.4. Electrochemical Impedance Spectroscopy (EIS) of Iridium(III) Dyes

**Figure 4:**
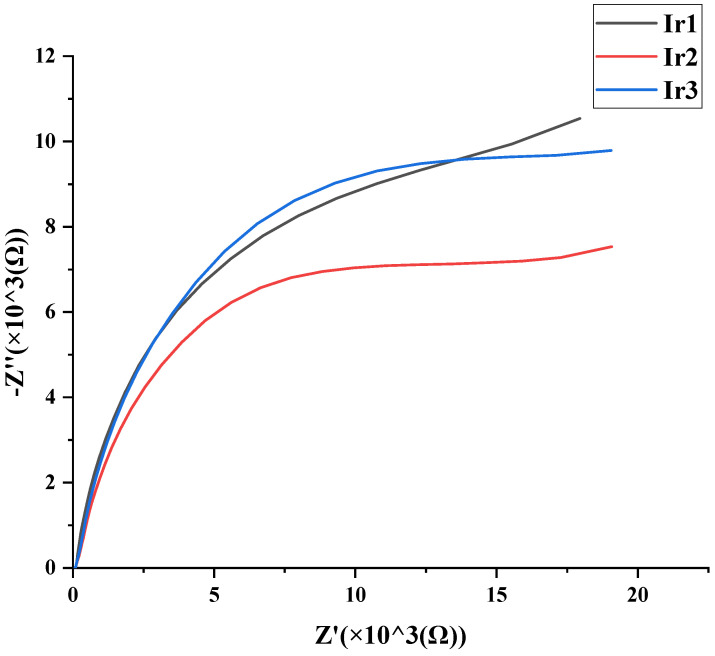
EIS Nyquist plots for complexes **Ir1**–**Ir3**.

To investigate the charge recombination properties of Ir(III) complexes, EIS was utilized [70,71]. Figure 4 shows the EIS Nyquist plots for **Ir1** to **Ir3**. It is well-established that a smaller arc radius on the EIS Nyquist plot indicates reduced resistance to electrical charge transfer, thereby enhancing hydrogen production performance [70,71,72,73]. **Ir2** demonstrated the smallest arc radius among the dyes, signifying its superior charge carrier transfer capabilities [74]. These observations are consistent with the hydrogen production efficiency of the dyes in water-splitting processes.

### 2.5. Photocurrent Measurements of Iridium(III) Dyes

**Figure 5:**
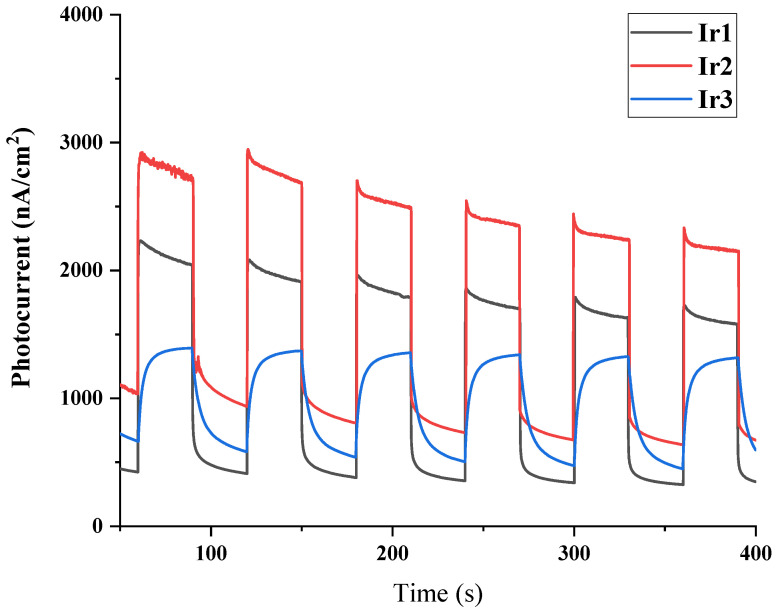
The photocurrent responses of **Ir1**–**Ir3**.

Photocurrent measurements were utilized to assess the stability and efficiency of charge separation in the metal complexes [75]. Consistent and rapid photocurrent response in light-on/light-off tests signifies steady photocatalytic activity [76] while heightened photocurrent density indicates efficient charge separation [77]. The photocurrent testing followed a previously established methodology [78]. Figure 5 illustrates the photocurrent behaviour of **Ir1**–**Ir3** under cycles of visible light exposure, depicting six on–off cycles. This clearly demonstrates efficient electron transfer [78], indicative of their consistent photocatalytic performance [76]. **Ir2** demonstrated a substantially increased photocurrent intensity when illuminated and a pronounced setback in its decrease upon light-off. This suggests greater efficiency in charge separation and an enhanced ability for hydrogen production compared to other dyes [76,78,79,80].

### 2.6. Electron Paramagnetic Resonance (EPR) Studies of ***Ir@Pt-TiO_2_***

**Figure 6:**
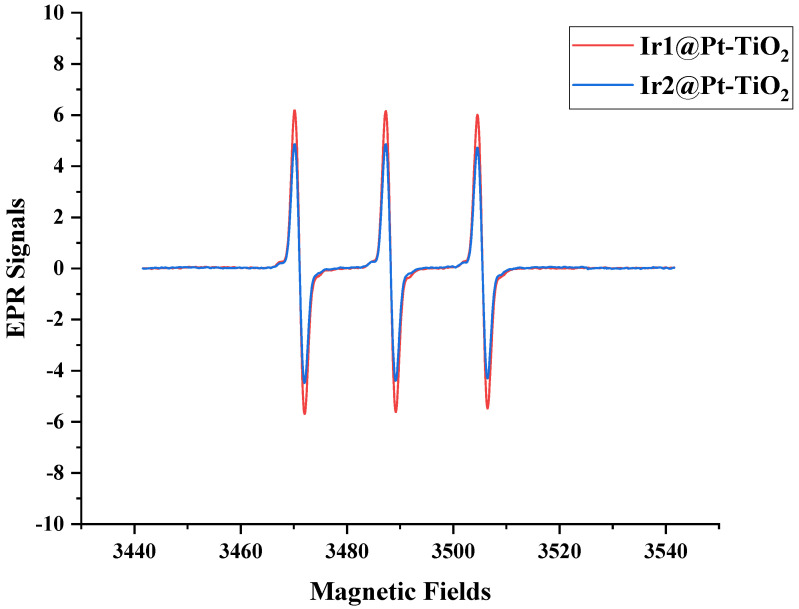
EPR results of **Ir1@Pt-TiO_2_** and **Ir2@Pt-TiO_2_**.

In this photocatalytic process, the photosensitizer is photoexcited under light irradiation, leading to electron transfer to the conduction band of TiO_2_. The photo-generated electrons and holes can undergo recombination in bulk or on the surface of the semiconductor very quickly, releasing energy in the form of heat or photons. These unreacted electrons are then relayed to Pt nanoparticles on the TiO_2_ surface, facilitating proton reduction and hydrogen release. Electron paramagnetic resonance was conducted to **Ir2@Pt-TiO_2_** and **Ir1@Pt-TiO_2_** (Figure 6). It is well known that as more electrons are produced, TEMPO^+^ is increasingly reduced, and as the amount of detectable TEMPO^+^ decreases, the signal decreases [81]. It can be inferred that, by observing the increased generation of photogenerated electrons by **Ir2@Pt-TiO_2_**, it is reasonable to anticipate that it will exhibit a higher efficiency in hydrogen production [82].

### 2.7. Light-Driven Hydrogen Generation Studies of ***Ir@Pt-TiO_2_***

The process of hydrogen generation through water splitting involved the use of Ir(III) complexes as photosensitizers. Detailed procedures for synthesizing platinized TiO_2_, conjugating Ir(III) complexes to the platinized TiO_2_, and executing the photocatalytic water-splitting process, are provided in the experimental section. Generally, each Ir(III) complex was bonded to the platinized TiO_2_ utilizing a sonication technique. Subsequently, the mixture underwent centrifugation and drying before the water-splitting experiment. Dye coupling effectiveness for each specimen was assessed by measuring absorbance shifts at the absorption peaks before and post-dye application, indicating a dyeing efficiency of approximately 100%.

**Figure 7:**
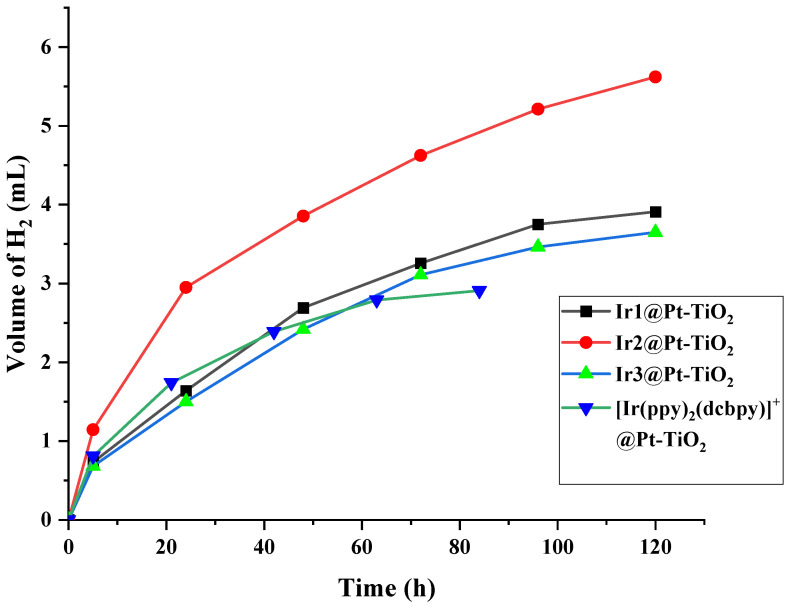
Photocatalytic H2 generation curves of **Ir1**–**Ir3@Pt-TiO_2_** and **[Ir(ppy)_2_(dcbpy)]^+^@TiO_2_** under blue LED irradiation (50 mW).

Photocatalytic water splitting for hydrogen generation was performed in a 5 mL AA solution (0.5 M) at pH 4.0, serving as the sacrificial electron donor. The experimental configuration employed single-neck 25 mL reaction flasks for the photocatalytic processes. Continuous illumination was provided by blue LEDs (470 nm). Hydrogen production from each specimen was qualitatively and quantitatively assessed using gas chromatography, with methane as the internal standard. Figure 7 illustrates the hydrogen production profiles over time for each sample. Table 3 presents relevant data, including TON, turnover frequency (TOF), initial turnover frequency (TOF_i_), and Activity_i_. In this photocatalytic process, the photosensitizer is photoexcited under light irradiation, leading to electron transfer to the conduction band of TiO_2_. These electrons are afterwards relayed to platinum nanoparticles on the TiO_2_ surface, facilitating proton reduction and hydrogen release. The oxidized photosensitizer is subsequently regenerated to its ground state by AA [36,57].

Among the tested systems, the **Ir2@Pt-TiO_2_** system demonstrated the most effective hydrogen generation ability, achieving TON values of 3670. Following closely, the **Ir1@Pt-TiO_2_** system recorded TON values of 2553. Higher hydrogen production was achieved when using Ir(III) dyes in the phosphonate anchoring group than in the carboxylate anchoring group. This improvement is attributed to the enhanced anchoring ability provided by the phosphonate group [44,83,84,85], a finding consistent with previous literature [45,51]. The photocatalytic water-splitting experiments for **[Ir(ppy)_2_(dcbpy)]Cl** were also performed. Figure 7 and Table 3 show the experimental results. The results suggest that our new complexes show higher stability than **[Ir(ppy)_2_(dcbpy)]Cl**. These findings indicate that our new Ir(III) dyes, especially with phosphonate anchoring group, are promising candidates for highly stable photocatalytic applications.

### 2.8. Toxicity Detection of ***Ir@Pt-TiO_2_***

**Figure 8:**
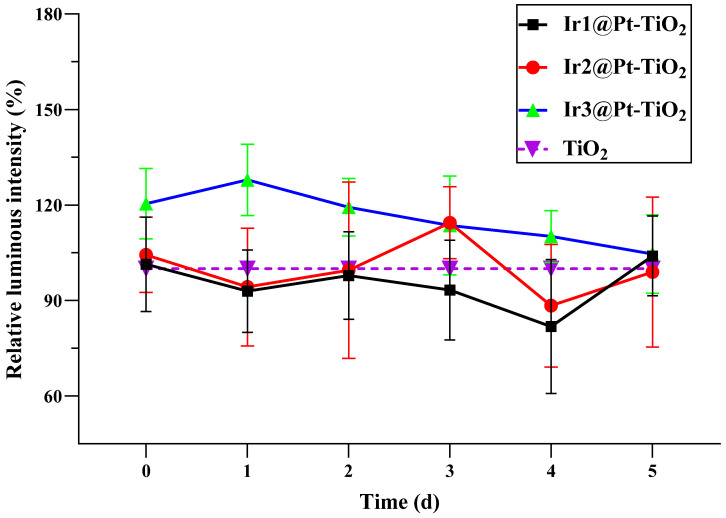
Relative luminous intensity of the photocatalytic materials. Data are expressed as the mean ± *SE* (*n* = 3). There was no significant difference (*p* > 0.05) in the toxicity of any of the experimental groups (Ir1@Pt-TiO_2_, Ir2@Pt-TiO_2_, and Ir3@Pt-TiO_2_) to luminescent bacteria compared to the control group (TiO_2_).

The luminous intensity of luminescent bacteria remains constant under specific conditions but changes upon contact with foreign substances. Within a defined concentration spectrum, the modulation of luminescent intensity correlates directly with toxin concentration. This characteristic facilitates the determination of overall toxicity through comparative assessment of luminous intensity pre- and post-exposure to a substance, employing a dedicated luminescent detector [86]. Several studies have suggested that the toxicity levels of materials can be evaluated through changes in the relative luminous intensity of luminescent bacteria [87,88,89]. The luminescent intensity was assessed subsequent to the amalgamation of photocatalytic materials with water samples containing luminescent bacilli T3 strain. Moreover, given titanium dioxide’s inherent antibacterial properties, it served as the control group in this study. This choice facilitated the computation of relative luminous intensity and enabled the comparative assessment of toxicity levels among various photocatalytic materials [90]. The photocatalytic materials (Ir1@Pt-TiO_2_, Ir2@Pt-TiO_2_, and Ir3@Pt-TiO_2_) were acquired following a photocatalytic process spanning 0 to 5 days, with subsequent incubation at room temperature for intervals of 0 and 15 min, as illustrated in Figure 8. With an increasing duration of the photocatalytic process, the mean relative luminous intensity of Ir3@Pt-TiO_2_ ranged from 104.6% to 120.4%, while for Ir1@Pt-TiO_2_ and Ir2@Pt-TiO_2_, it ranged from 81. 8% to 114.4%. However, no noteworthy alterations were discerned in the relative luminous intensity of the luminescent bacteria when exposed to the photocatalytic materials (**Ir1@Pt-TiO_2_**, **Ir2@Pt-TiO_2_**, and **Ir3@Pt-TiO_2_**), relative to those exposed to TiO_2_. Traditionally, TiO_2_ particles have been viewed as having low solubility and minimal toxicity [91]. These findings suggest that the photocatalytic materials (**Ir1@Pt-TiO_2_**, **Ir2@Pt-TiO_2_**, and **Ir3@Pt-TiO_2_**) do not induce significant toxic effects within the specified timeframe. It is noteworthy that iridium complexes, due to their low toxicity, are commonly utilized in biology and life sciences [92]. Ajay et al. [93] found that iridium exhibits variable oxidation states and dynamic stability in biological systems, making it a viable option as an anticancer drug. Consequently, the photocatalytic materials (**Ir1@Pt-TiO_2_**, **Ir2@Pt-TiO_2_**, and **Ir3@Pt-TiO_2_**) utilized in this investigation do not pose an elevated risk to the aquatic environment compared to TiO_2_.

## 3. Materials and Methods

### 3.1. Materials and Reagents

All the reactions were carried out under a nitrogen atmosphere with the standard Schlenk technique. All the glassware were dried in the oven overnight before use. All the solvents were dried by distillation with appropriate drying agents under an N_2_ atmosphere. All the reagents for chemical synthesis were purchased from Sigma-Aldrich or Dieckmann. Apart from those specifically stated, all the chemicals were directly used as received. All the reactions were monitored by thin-layer chromatography (TLC) with Merck silica gel pre-coated aluminum plates. Purification of the products were achieved by column chromatography using silica gel (230–400 mesh) or basic aluminum oxide purchased from Dieckmann. **1-(furan-2-yl)isoquinoline** was synthesized according to a previous report [94]. Details of experiments can be found in the Supplementary Materials.

### 3.2. Synthesis of Materials

**1-(5-bromofuran-2-yl)isoquinoline:** To a round bottom flask containing 1-(furan-2-yl)isoquinoline (2.470 g, 12.630 mmol) in dichloromethane (10 mL), n-bromosuccinimide (2.701 g, 15.160 mmol) was added slowly. The reaction mixture was then stirred at 293 K overnight. The mixture was extracted with ethyl acetate and brine. The organic layer was dried over sodium sulfate, filtered, and concentrated by reduced pressure. The crude product was purified by silica gel column chromatography using dichloromethane/*n*-hexane (1:1, *v*/*v*) as eluent to give the final product as a yellow oil (yield: 3.121 g, 90%). ^1^H NMR (600 MHz, CDCl_3_) δ 8.68 (d, *J* = 8.5 Hz, 1H), 8.53 (d, *J* = 5.4 Hz, 1H), 7.81 (d, *J* = 8.1 Hz, 1H), 7.69–7.60 (m, 2H), 7.57 (d, *J* = 5.6 Hz, 1H), 7.14 (d, *J* = 3.1 Hz, 1H), 6.55 (d, *J* = 3.1 Hz, 1H). ^13^C NMR (151 MHz, CDCl_3_) δ 155.7, 147.8, 142.1, 137.1, 130.2, 128.0, 127.2, 126.3, 125.3, 123.7, 120.6, 114.8, 113.7. Found: [M + H]^+^ 273.9869; ‘molecular formula C13H8BrNO’ requires [M + H]^+^ 273.9862.

**L1:** To a round bottom flask containing 1-(5-bromofuran-2-yl)isoquinoline (4.110 g, 25.120 mmol) in tetrahydrofuran (250 mL), (9-phenyl-9H-carbazol-3-yl) boronic acid (4.220 g, 37.680 mmol) was added. Tetrakis(triphenylphosphine) palladium(0) (2.902 g, 2.512 mmol) and 2 M of potassium carbonate (76 mL, 150.720 mmol) were added to the reaction mixture, which was then heated to 85 °C for 48 h. After being cooled to room temperature, the mixture was extracted with ethyl acetate and brine. The organic layer was dried over sodium sulfate, filtered, and concentrated by reduced pressure. The crude product was purified by silica gel column chromatography using dichloromethane/n-hexane (1:1, *v*/*v*) as eluent to give the final product as a yellow solid (yield: 4.102 g, 84%). ^1^H NMR (600 MHz, DMSO) δ 9.05–8.96 (m, 1H), 8.79 (d, *J* = 1.8 Hz, 1H), 8.60 (d, *J* = 5.5 Hz, 1H), 8.41 (d, *J* = 7.7 Hz, 1H), 8.12–7.98 (m, 2H), 7.93–7.79 (m, 3H), 7.76–7.64 (m, 4H), 7.59 (tt, *J* = 7.1, 1.4 Hz, 1H), 7.54–7.45 (m, 3H), 7.42 (d, *J* = 8.2 Hz, 1H), 7.39–7.34 (m, 1H), 7.28 (d, *J* = 3.5 Hz, 1H). ^13^C NMR (151 MHz, DMSO) δ 156.2, 153.1, 148.2, 142.7, 141.2, 140.4, 137.3, 137.0, 130.9, 130.8, 128.9, 128.4, 127.9, 127.3, 127.2, 126.5, 124.9, 123.7, 123.2, 123.1, 122.9, 121.5, 120.9, 120.5, 116.7, 115.9, 110.9, 110.4, 107.2. Found: [M + H]^+^ 437.1665; ‘molecular formula C_31_H_20_N_2_O’ requires [M + H]^+^ 437.1648.

**Ir1**: To a round bottom flask containing **L1** (1.000 g, 2.291 mmol), iridium(III) chloride hydrate (0.270 g, 0.764 mmol) was added with 2-ethoxyethanol/deionized water (3:1, *v*/*v*, total 8 mL). The reaction mixture was heated at 90 °C for 20 h. The reagent was purified by filtration to give the yellow solid as the iridium dimer **Ir_2_L1_4_Cl_2_**. This compound was used in the subsequent reaction without further purification.

To a round bottom flask containing iridium dimer **Ir_2_L1_4_Cl_2_** (0.400 g, 0.182 mmol) in dichloromethane/methanol (1:1, *v*/*v*, total 6 mL), diethyl [2,2′-bipyridine]-4,4′-dicarboxylate (0.137 g, 0.455 mmol) was added. The reaction mixture was then heated to 65 °C for 6 h. After cooling to room temperature, the pH was adjusted to 5 by introducing an appropriate amount of 1 M HCl. The precipitate was filtered. The crude product was purified by silica gel column chromatography using dichloromethane/methanol (1:1, *v*/*v*) as eluent to give the final product as a yellow solid (yield: 0.089 g, 36%). ^1^H NMR (600 MHz, CDCl_3_) δ 9.41–9.17 (m, 2H), 9.06–8.87 (m, 2H), 8.70–8.54 (m, 2H), 8.33 (d, *J* = 9.3 Hz, 2H), 8.25–8.07 (m, 4H), 7.94–7.68 (m, 9H), 7.68–7.38 (m, 20H), 7.38–7.30 (m, 5H). Found: [M + NH_4_]^+^ 1325.3530; ‘molecular formula C_74_H_46_IrN_6_O_6_’ requires [M + NH_4_]^+^ 1325.3453

**Ir2**: To a round bottom flask containing iridium dimer **Ir_2_L1_4_Cl_2_** (0.200 g, 0.091 mmol) in dichloromethane/methanol (1:1, *v*/*v*, total 6 mL), tetraethyl [2,2′-bipyridine]-4,4′-diylbis(phosphonate) (0.097 g, 0.227 mmol) was added. The reaction mixture was then heated to 65 °C for 6 h. After cooling to room temperature, the pH was adjusted to 5 by introducing an appropriate amount of 1 M HCl. The precipitate was filtered. The crude product was purified by silica gel column chromatography using dichloromethane/methanol (1:1, *v*/*v*) as eluent to give the final product as a yellow solid (yield: 0.052 g, 39%). ^1^H NMR (600 MHz, CDCl_3_) δ 8.86–8.72 (m, 1H), 8.71–8.54 (m, 1H), 8.14 (d, *J* = 8.1 Hz, 3H), 7.98–7.69 (m, 8H), 7.69–7.51 (m, 14H), 7.51–7.29 (m, 17H), 4.34–4.12 (m, 8H), 1.03–0.62 (m, 12H). Found: [M]^+^ 1491.3888; ‘molecular formula C_80_H_64_IrN_6_O_8_P_2_’ requires [M]^+^ 1491.3893.

**Ir3**: To a round bottom flask containing iridium dimer **Ir_2_L1_4_Cl_2_** (0.200 g, 0.091 mmol) in dichloromethane/methanol (1:1, *v*/*v*, total 6 mL), [2,2′-Biquinoline]-4,4′-dicarboxylic acid (0.080 g, 0.227 mmol) was added. The reaction mixture was then heated to 65 °C for 6 h. After cooling to room temperature, the pH was adjusted to 5 by introducing an appropriate amount of 1 M HCl. The precipitate was filtered. The crude product was purified by silica gel column chromatography using dichloromethane/methanol (1:1, *v*/*v*) as eluent to give the final product as a yellow solid (yield: 0.044 g, 33%). ^1^H NMR (600 MHz, CDCl_3_) δ 8.61 (d, *J* = 9.6 Hz, 2H), 8.32 (d, *J* = 1.7 Hz, 1H), 8.25–8.05 (m, 4H), 7.98–7.77 (m, 6H), 7.80–7.66 (m, 6H), 7.68–7.46 (m, 18H), 7.45–7.27 (m, 13H). Found: [M]^+^ 1407.3763; ‘molecular formula C_82_H_50_IrN_6_O_6_’ requires [M]^+^ 1407.3424.

## 4. Conclusions

This study introduces novel Ir(III) photosensitizers incorporating 9-phenyl-9H-carbazole, featuring either the phosphate linker or carboxylic acid anchoring groups. Each Ir(III) dye underwent thorough characterization and assessment of hydrogen generation rates through water splitting. Analysis of the UV–Vis absorption spectra of the Ir(III) dyes revealed significantly heightened intensities extending into the visible region, especially notable in dyes containing isoquinoline functional groups. This enhancement bolstered their light-harvesting ability and consequently improved hydrogen production.

The water splitting tests revealed that the **Ir2@Pt-TiO_2_** system, featuring the Cz and isoquinoline groups with a phosphate anchoring group, achieved the highest TON of 3670 under blue LED irradiation. This finding underscores the advantageous influence of 9-phenyl-9H-carbazole and isoquinoline, attributed to their strong intramolecular charge transfer ability. In addition, the Ir(III) dye systems employing the phosphate anchoring group demonstrated superior TON values than those with identical C^N ligands but utilizing carboxylic acid. Therefore, the phosphate anchoring group is crucial for designing highly effective photosensitizers with exceptional stability. Toxicological studies were concurrently conducted on three iridium(III) complexes and TiO_2_. The findings revealed minimal or negligible differences in luminous intensity among them. Furthermore, the results indicated that iridium(III) complexes do not pose an elevated risk to the aquatic environment compared to TiO_2_.

## Figures and Tables

**Table 1:** UV–Vis absorption parameter values of **Ir1** to **Ir3** in CH_2_Cl_2_ at 293 K.

Dye	*λ*_max_/nm (*ε*/10^5^ M^−1^ cm^−1^)	*λ*_onset_/nm
**Ir1**	234 (5.45), 280 (2.64), 490 (0.96)	597
**Ir2**	233 (5.36), 278 (2.64), 306 (2.43), 497 (1.07)	574
**Ir3**	237 (4.91), 283 (2.51), 492 (0.84)	601
**[Ir(ppy)_2_(dcbpy)]** **Cl**	240 (2.64), 311 (0.86), 340 (0.52), 398 (0.12)	427

**Table 2:** Electrochemical data and energy levels of **Ir1**–**Ir3**.

Dye	$E_{Ox}^{Max}$/V	*E*_HOMO_ ^[a]^/eV	*E*_g_ ^[b]^/eV	*E*_LUMO_ ^[c]^/eV
**Ir1**	0.86	−5.66	2.07	−3.59
**Ir2**	0.85	−5.65	2.16	−3.49
**Ir3**	0.86	−5.66	2.06	−3.60
**[Ir(ppy)_2_(dcbpy)]Cl**	1.39	−6.19	2.90	−3.29

^[a]^ Calculated as −($E_{Ox}^{Max}$ + 4.8). ^[b]^ Energy band gap, determined from the onset of absorption. ^[c]^ *E*_LUMO_ = *E*_HOMO_ + *E*_g_.

**Table 3:** Photocatalytic H_2_ generation data for different Ir(III) dyes attached to platinized TiO_2_ (**Ir1–Ir3@Pt-TiO_2_**) under blue light irradiation.

Dye	Time/h	H_2_/mL	TON ^[a]^	TOF ^[b]^/h^−1^	TOF_i_ ^[c]^/h^−1^	Activity_i_ ^[d]^/µmol g^−1^ h^−1^
**Ir1**	120	3.91	2553	21.3	95.7	59,810
**Ir2**	120	5.62	3670	30.6	149.4	93,387
**Ir3**	120	3.85	2383	19.9	89.0	55,640
**[Ir(ppy)_2_** **(dcbpy)]^+^**	84	2.91	1900	22.6	105.8	66,122

^[a]^ TON for hydrogen production was determined by doubling the moles of hydrogen generated compared to the moles of photosensitizer bonded to the platinized TiO_2_. ^[b]^ The TOF of the system was calculated on an hourly basis. ^[c]^ Initial turnover frequency (TOF_i_) was measured during the first 5 h of the reaction. ^[d]^ Activityi is described as hydrogen quantity (in micromoles) evolved per hour per gram of platinum utilized.

## Data Availability

The data presented in this study are available on request from the corresponding author.

## Supplementary Materials

The following are available online at https://www.mdpi.com/article/10.3390/molecules29112564/s1, Figure S1: ^1^H NMR spectrum of **1-(furan-2-yl)isoquinoline** in CDCl_3_, Figure S2: ^13^C NMR spectrum of **1-(furan-2-yl)isoquinoline** in CDCl_3_, Figure S3: ^1^H NMR spectrum of **1-(5-bromofuran-2-yl)isoquinoline** in CDCl_3_,Figure S4: ^13^C NMR spectrum of **1-(5-bromofuran-2-yl)isoquinoline** in CDCl_3_, Figure S5: ^1^H NMR spectrum of **L1** in CDCl_3_, Figure S6: ^13^C NMR spectrum of **L1** in CDCl_3_, Figure S7: ^1^H NMR spectrum of **Ir1** in CDCl_3_, Figure S8: ^1^H NMR spectrum of **Ir2** in CDCl_3_, Figure S9: ^13^C NMR spectrum of **Ir3** in CDCl_3_, Figure S10: CV results of (a) **Ir1**, (b) **Ir2**, (c) **Ir3**, and (d) **[Ir(ppy)2(dcbpy)]Cl**, Figure S11: calibration plot of the integrated amount of hydrogen relative to the methane, Figure S12: FTIR spectrum of **Ir1**, Figure S13: FTIR spectrum of **Ir2**, Figure S14: FTIR spectrum of **Ir3**, Figure S15: XPS results of **Ir1@Pt-TiO_2_**, Figure S16: XPS results of **Ir2@Pt-TiO_2_**, Figure S17: XPS results of **Ir3@Pt-TiO_2_**, Figure S18: The emission spectra of used LEDs. Table S1. FTIR spectral analysis of **Ir1**, Table S2. FTIR spectral analysis of **Ir2**, Table S3. FTIR spectral analysis of **Ir3**. Refs. [95,96,97,98,99,100,101,102,103,104,105,106] are cited in Supplementary Materials file.
