# Supplementary material for: Impact of Anchoring Groups on the Photocatalytic Performance of Iridium(III) Complexes and Their Toxicological Analysis
Source: Molecules. 2024 May 30;29(11):2564. doi: 10.3390/molecules29112564 (PMC11173709; doi:10.3390/molecules29112564)
Supplement: Supplementary file 1 [file molecules-29-02564-s001.zip › molecules-2994474-supplementary.pdf]

Impact of Anchoring Groups on the Photocatalytic Performance of Iridium(III) Complexes and Their Toxicological Research

Instrumentation

Matrix-assisted laser desorption Ionization Time-of-Flight (MALDI-TOF) or Liquid Chromatography–Electrospray Ionization Quadrupole-TOF (LC-ESI-Q-TOF) mass spectrometry was performed on an Autoflex Bruker MALDI-TOF or Agilent 6540 system, respectively. ^{1}H and ^{13}C NMR spectra were measured in $CDCl_3$ on a Bruker Ultra-shield 400 MHz FT-NMR spectroscopy and tetramethylsilane (TMS) was used as an internal standard for calibrating the chemical shift. UV/Vis absorption spectra were recorded on an Agilent Technologies Cary 8454 UV-Vis spectrometer in CH_2Cl_2 solution at 293 K. PL spectra were recorded on a Cary Eclipse Fluorescence Spectrophotometer in CH_2Cl_2 solution at 293 K. ERP was conducted by Guoyi Quantum 200. Cyclic voltammetry (CV) measurements were performed with a CHI 680D Electrochemical Analyzer/Workstation, using glassy carbon electrode as the working electrode, platinum wire as the counter electrode, and Ag/Ag^{+} as the reference electrode, in acetonitrile solution containing 0.1 M tetrabutylammonium hexafluorophosphate at a scan rate of 100 mV s^{-1}. Under these conditions, the reversible oxidation potential ($E_{1/2}$) of ferrocene was 0.40 V versus Ag/Ag^{+}. XPS was conducted on Thermo SCIENTIFIC ESCALAB 250Xi. Fourier-transform infrared (FT-IR) characterization were determined by Bruker Vector22 infrared spectroscopy at room temperature, and KBr was used as a blank control.

Synthesis of Materials:

1-(furan-2-yl)isoquinoline : To a round bottom flask containing 1-chloroisoquinoline (4.110 g, 25.120 mmol) in tetrahydrofuran (250 mL), furan-2-ylboronic acid (4.220 g, 37.680 mmol) was added. Tetrakis(triphenylphosphine)palladium(0) (2.902 g, 2.512 mmol) and 2M of potassium carbonate (76 mL, 150.720 mmol) were added to the reaction mixture, which was then heated to 115 °C for 48 h. After being cooled to room temperature, the mixture was extracted with ethyl acetate and brine. The organic layer was dried over sodium sulfate, filtered and concentrated by reduced pressure. The crude product was purified by silica gel column chromatography using dichloromethane/n-hexane (1:1, v/v) as eluent to give the final product as a yellow solid (yield: 4.102 g, 84%). ^{1}H NMR (600 MHz, $CDCl_3$) δ 8.74 (d, J = 8.5 Hz, 1H), 8.61 – 8.49 (m, 1H), 7.85 – 7.75 (m, 1H), 7.72 – 7.51 (m, 4H), 7.18 (d, J = 2.7 Hz, 1H), 6.62 (d, J = 1.6 Hz, 1H). ^{13}C NMR (101 MHz, CDCl3) δ 153.8, 149.0, 143.9, 142.2, 137.1, 130.0, 127.7, 127.2, 126.7, 125.5, 120.2, 112.7, 111.8. Found: $[M+Na]^+$ 218.0607; 'molecular formula $C_{13}H_9NO$ ' requires $[M+Na]^+$ 218.0576.

Cyclic voltammetry measurements

Cyclic voltammetry (CV) experiments were conducted with a CHI 680D electrochemical analyser using glassy carbon as the working electrode, Pt wire as the counter electrode and Ag/Ag^+ as the reference electrode using dichloromethane comprising 0.1 M tetrabutylammonium hexafluorophosphate as the solvent at a scan rate of 100 mV s^{-1}. Under these conditions, the reversible oxidation potential ($E_{1/2}$) of

ferrocene was 0.40 V versus Ag/Ag^+[1].

Electrochemical impedance spectroscopy and photocurrent measurements

Electrochemical impedance spectroscopy (EIS) was performed on an electrochemical workstation CHI660E (Shanghai Chenhua, China), using a three-electrode system comprising Ag/AgCl as the reference electrode and Pt wire as the counter electrode, with 0.5 M sodium sulphate (Na_2SO_4) solution as electrolyte. To prepare the working electrode, 10 mg of the iridium(III) dyes was dispersed in 1 mL ethanol and 20 μL Nafion aqueous solution (5 wt%) and then ultrasonically scattered for 2 h. Subsequently, 0.1 mL of the slurry was added dropwise onto an FTO glass substrate (1 cm^2). The iridium(III) dye remained coated on the FTO glass surface after the ethanol had been evaporated off. EIS was conducted in the frequency range of 0.1 Hz to 100 kHz at room temperature with the applied bias potential set at –700 mV and the alternate current amplitude set at 5 mV. Photocurrent spectra were obtained in the frequency range of 0.1 Hz to 100 kHz with an applied bias potential of –700 mV and an AC amplitude of 5 mV. Light was supplied via a 300 W Xe lamp. All the experiments were conducted at room temperature.

Preparation of platinized TiO_2

To a one-neck round bottom flask containing 1.6 g of titanium oxide powder (anatase, < 25 nm particle size, 99.7% trace metals basis, Sigma-Aldrich) and 0.1 mL of H_2PtCl_6 aqueous solution (8 wt%), 40 mL MeOH was added. The reaction mixture was then

subjected to radiation by a 300 W Hg lamp (HF300PD, EYE Lighting) for 24 h with stirring. The mixture was undergone centrifugation at 4000 rpm for 5 mins and the solid obtained was washed with MeOH three times. The remaining solid was dried under vacuum in darkness overnight.

Adsorption of iridium(III) photosensitizer onto platinized TiO_2

To a centrifuge tube containing 20 mg of Pt-TiO_2, 2.5 mL of 50 μM photosensitizer in MeOH solution was added, and the mixture was then sonicated for 30 mins. The solution was decolorized and became clear gradually, while the grey color solid was changed to light pink. The tube was centrifuged at 4000 rpm for 15 mins and the liquid layer was removed by a dropper. The remaining solid was dried in darkness overnight. The whole dried pellet was directly utilized in the photocatalytic reaction. The dye-loading percentage was estimated by comparing the absorbance of the absorption peaks between the supernatant and the original photosensitizer solution at low energy region.

Light-driven H_2 production studies

To a one-neck pear-shaped round bottom flask containing the dye-adsorbed platinized TiO_2, 5 mL of 0.5 M of AA (pH = 4) was added as the hole scavenger. The flask was then sealed by rubber septa and was purged with a mixture of argon/methane (80:20 mol%) for 15 mins. The methane present in the gas mixture was served as an internal standard for GC analysis of each experiment. The flask was steadily stirred and continuously radiated from the bottom with blue (*ca.* 470 nm) light-emitting diodes

inside a just-fit container, which blocks the stray light from the environment. The light power was measured using a thermal sensor and power meter (Model: BIM-7203-0100F & BIM-7001; Hangzhou Brolight Technology Co., Ltd.) and estimated to be 50 mW for each reaction. The produced hydrogen was measured by GC (Agilent 6890 Series GC System with a molecular sieve 5 Å column and thermal conductivity detector) at different time points from the headspace of reaction mixtures and was quantified using a calibration plot of the integrated amount of hydrogen relative to the methane (**Fig. S11**). The LED radiation is assumed to be monochromatic at emission intensity maximum (470 nm for blue LEDs).

Toxicity Testing

To investigate the toxicity of intermediate and final products generated by photocatalytic materials (**Ir1@Pt-TiO_2**, **Ir2@Pt-TiO_2**, **Ir3@Pt-TiO_2**) in water samples during the process of photocatalytic hydrogen production, the toxicity of the photocatalytic materials' water samples was assessed after a photocatalytic process lasting 0-5 days. This evaluation was conducted in accordance with the international standards (ISO11348: 2007) and the national standards of the People's Republic of China (GB/T15441-1995), using Bacillus luminiformis T3 as the indicator organism[2]. This study employed the Luminescent bacterium Photobacterium T3 race as the test species to measure the biotoxicity of the photocatalytic materials' water samples before and after the photocatalytic process.

In the experiment, 190 μL of photocatalytic materials water samples collected at

different sampling times were transferred into a 96-well plate. As a control, 190 μL of a 3% NaCl sterile solution was added. Subsequently, 10 μL of Luminescent bacilli for detection were introduced into the water samples. The 96-well plate was then positioned on the detector of a chemiluminescence reading instrument, with a detection time set to 100 ms. The water samples in the 96-well plate were allowed to stand for 0 and 15 mins, and the luminescence intensity of each well was measured. The stability of the luminescent bacteria was inferred when the relative luminous intensity value of the bacterial liquid in the control group fell within the range of 0.6-1.8. The calculation method can be expressed using the following equations (Eq. S1):

$$C_f = \frac{C_{15}}{C_0} \quad (S1)$$

C_f: the relative luminous intensity value of the bacterial liquid in the control group;

C_{15}: the intensity of the glowing bacteria after 15 mins;

C_0: the luminous intensity of the bacteria at 0 mins.

The relative luminous intensity (R%) was calculated as the ratio of the luminescence intensity of the sample to the luminescent bacteria, as shown in Eq. S2. To ensure the reproducibility of the biotoxicity data, the acute toxicity assays were conducted in triplicate in this study.

$$R\ (\%) = \frac{M_{15}}{T_{15}} \times 100\% \quad (S2)$$

R (%): The relative luminous intensity of the luminescence intensity of the sample;

M_{15}: The luminous intensity of the photocatalytic materials **Ir1@ Pt-TiO_2**, **Ir2@ Pt-TiO_2**, and **Ir3@Pt-TiO_2** at 15 mins;

T_{15}: The luminous intensity of TiO_2 at 15 mins.

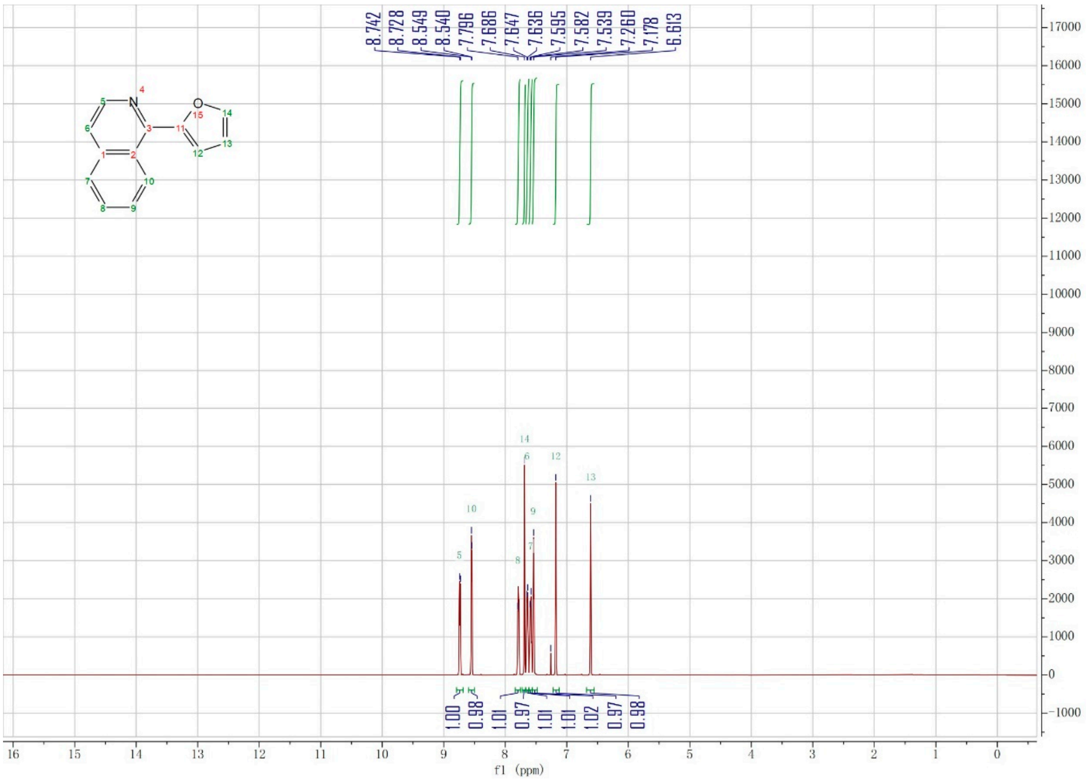

Figure S1: ^{1}H NMR spectrum of **1-(furan-2-yl)isoquinoline** in $CDCl_3$

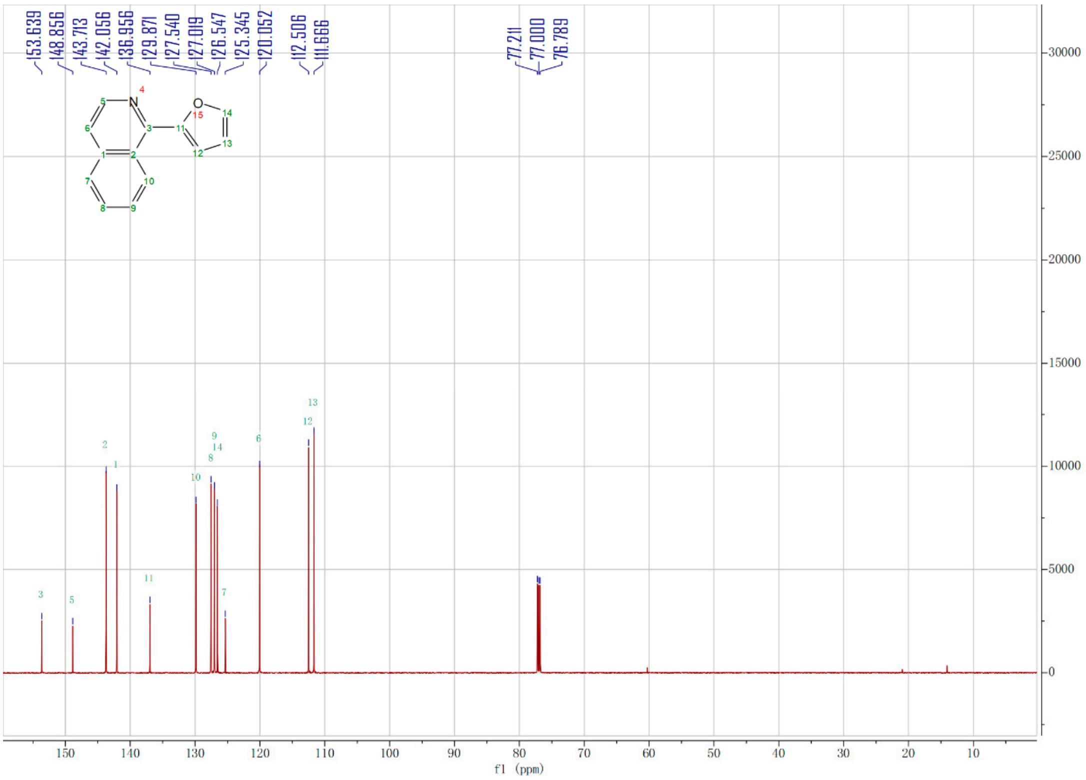

Figure S2: ^{13}C NMR spectrum of **1-(furan-2-yl)isoquinoline** in $CDCl_3$

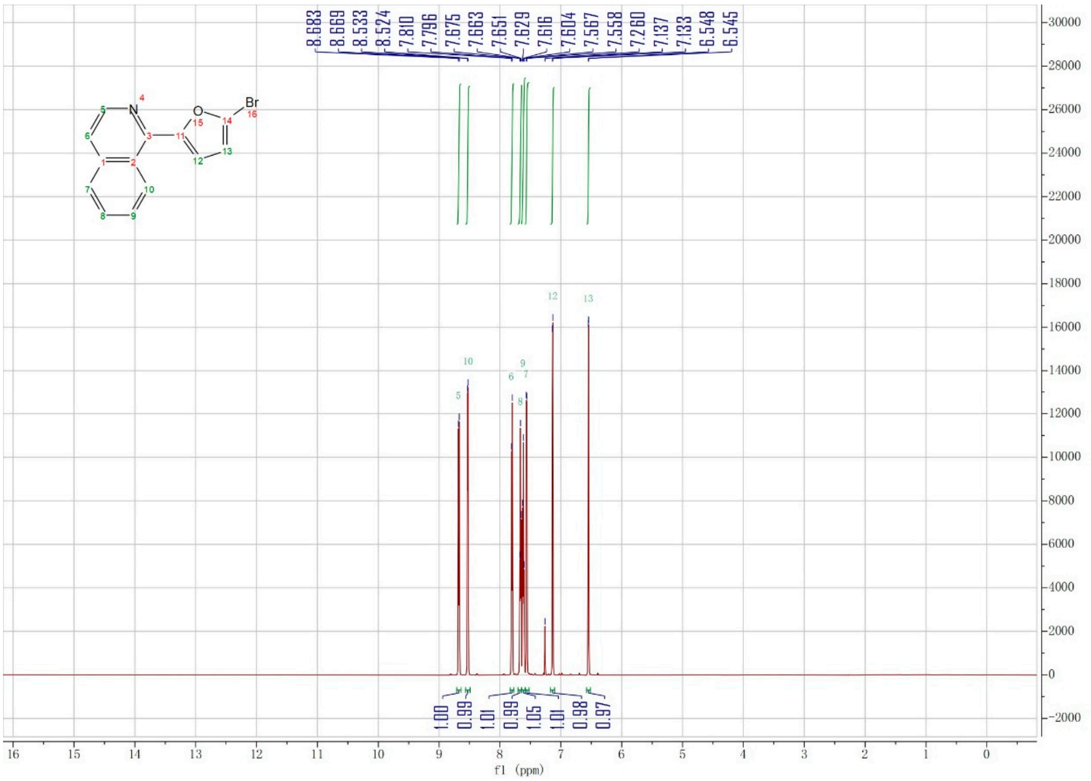

Figure S3: ^{1}H NMR spectrum of **1-(5-bromofuran-2-yl)isoquinoline** in $CDCl_3$

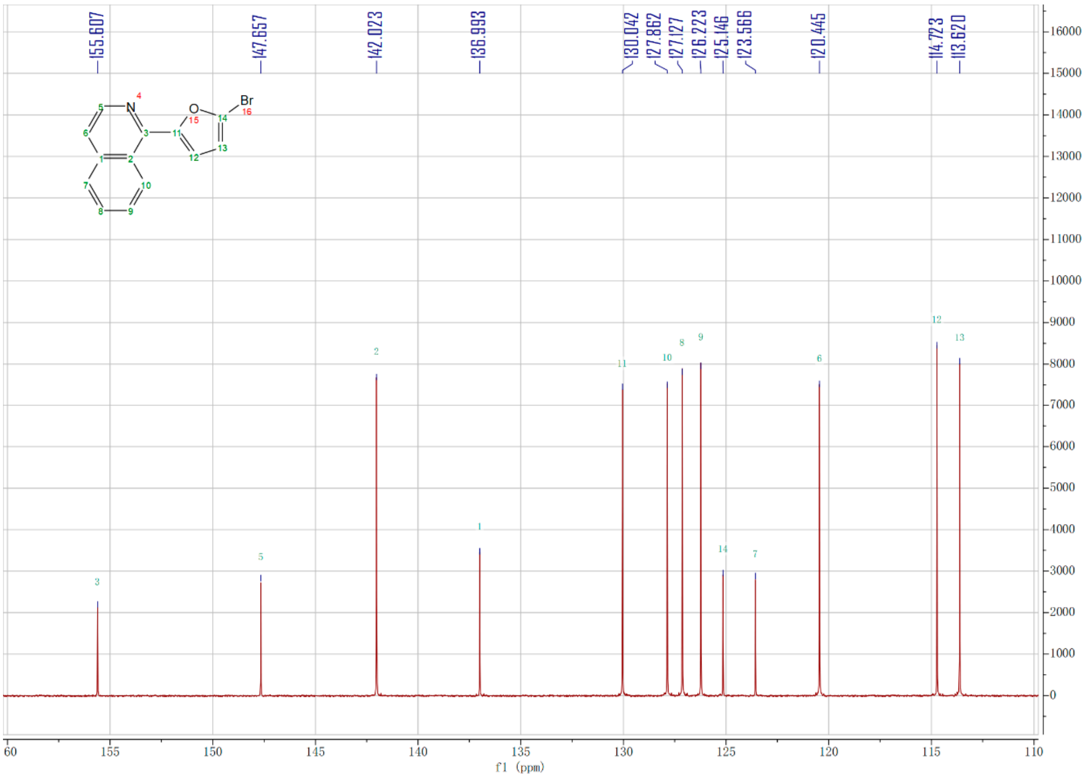

Figure S4: ^{13}C NMR spectrum of **1-(5-bromofuran-2-yl)isoquinoline** in $CDCl_3$

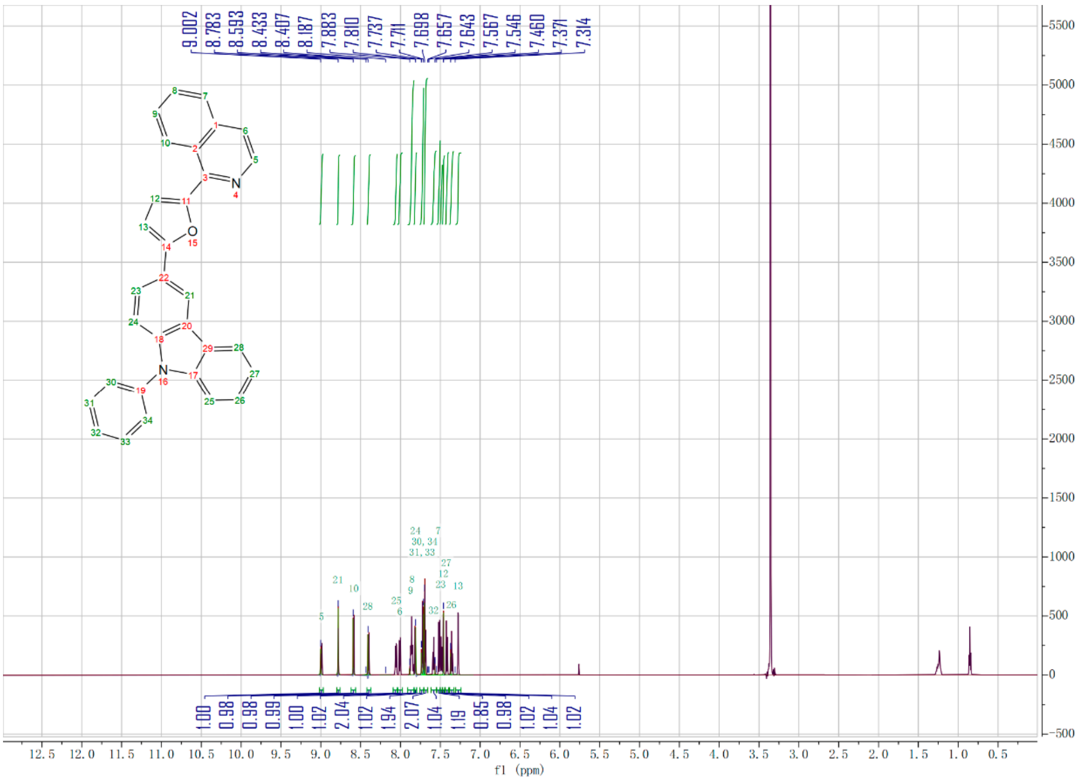

Figure S5: ^{1}H NMR spectrum of **L1** in $CDCl_3$

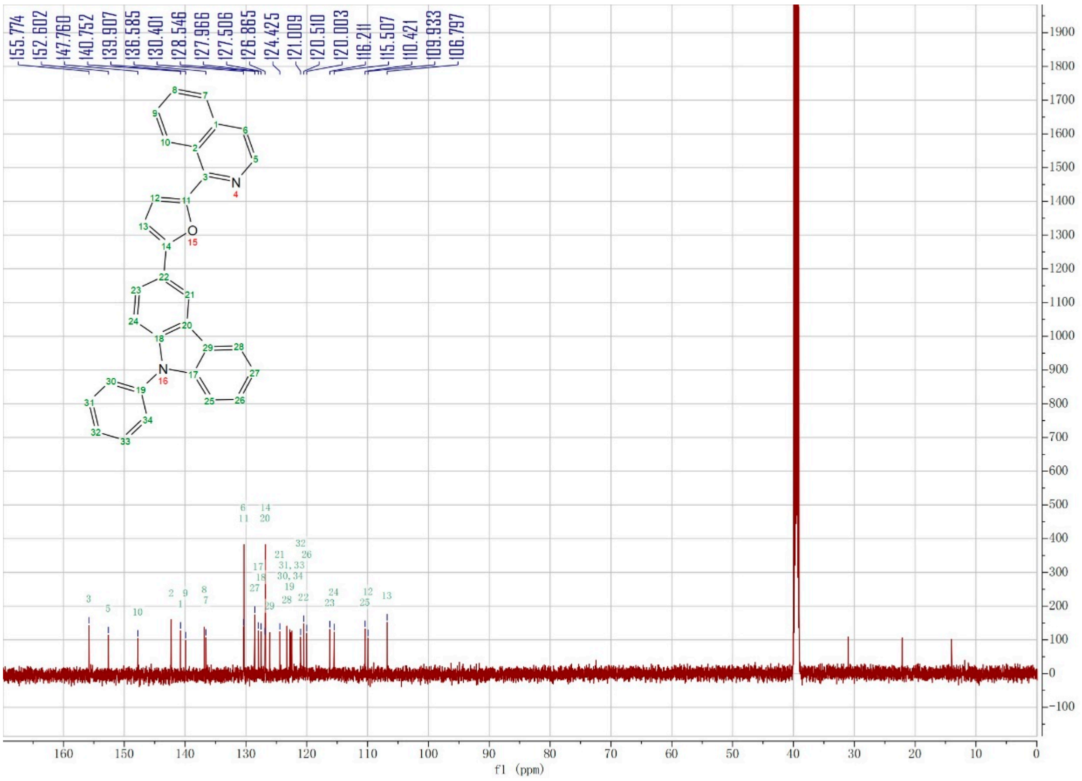

Figure S6: ^{13}C NMR spectrum of **L1** in $CDCl_3$

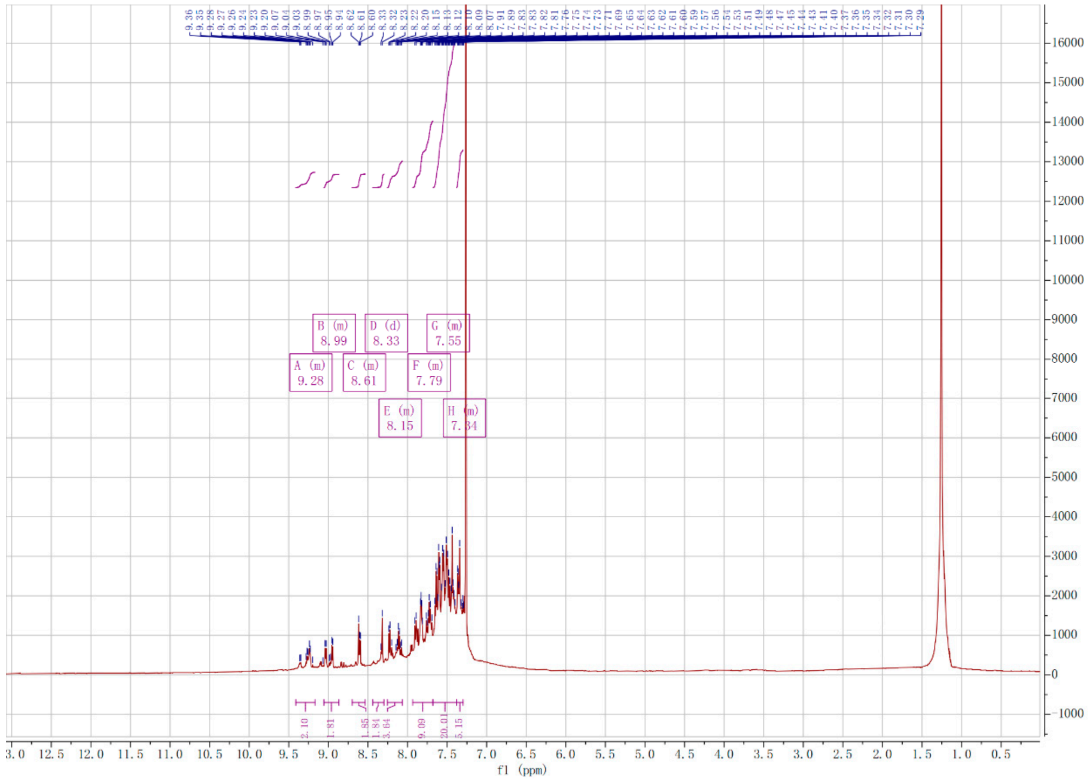

Figure S7: ^{1}H NMR spectrum of **Ir1** in $CDCl_3$

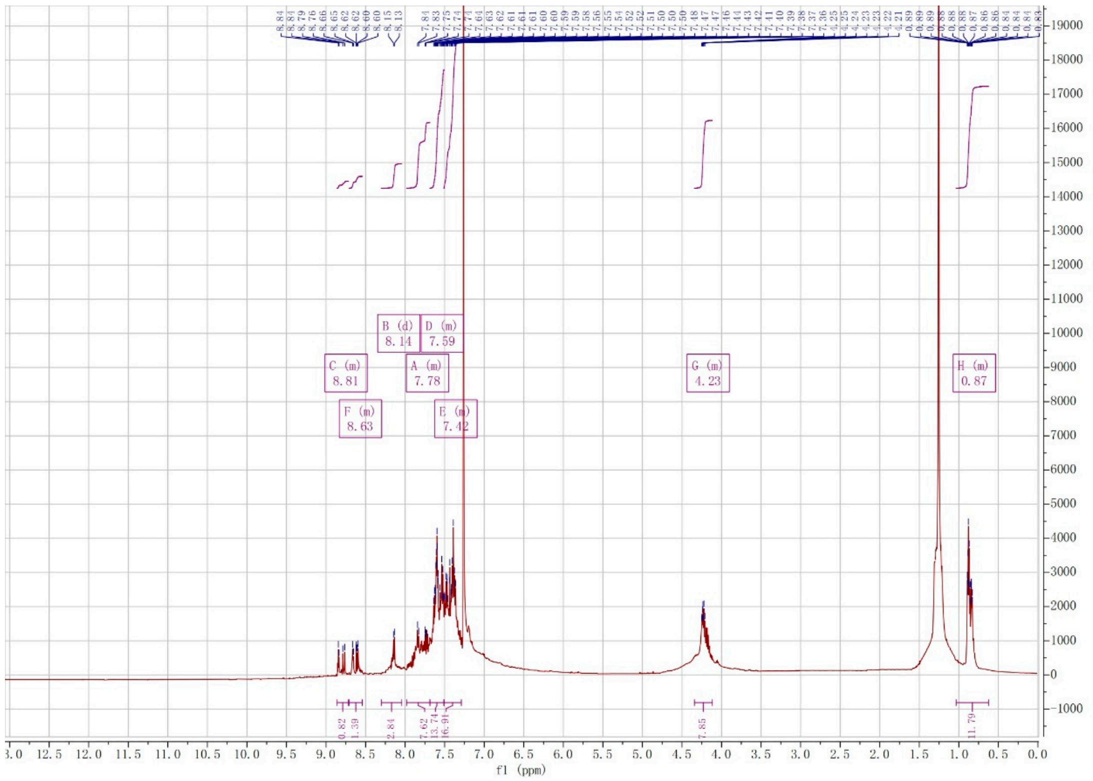

Figure S8: ^{1}H NMR spectrum of **Ir2** in $CDCl_3$

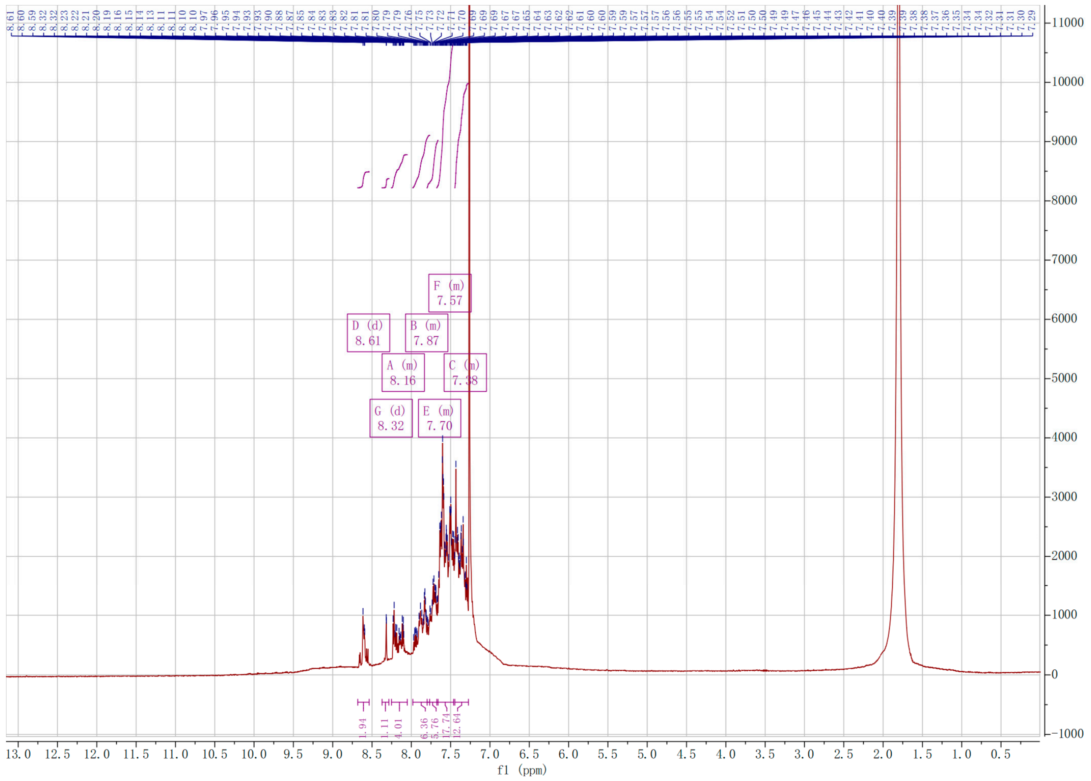

Figure S9: ^{13}C NMR spectrum of **Ir3** in $CDCl_3$

a

b

c

d

Figure S10: CV results of (a) **Ir1**, (b)**Ir2**, (c)**Ir3**, and (d) **[Ir(ppy)$_2$(dcbpy)]Cl**

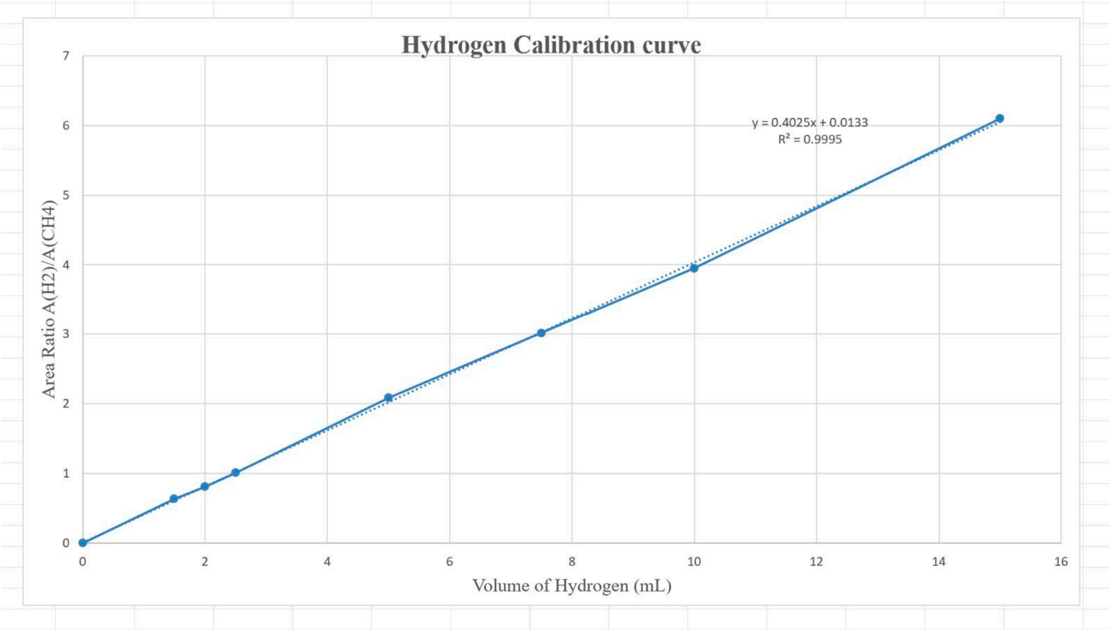

Figure S11: calibration plot of the integrated amount of hydrogen relative to the methane

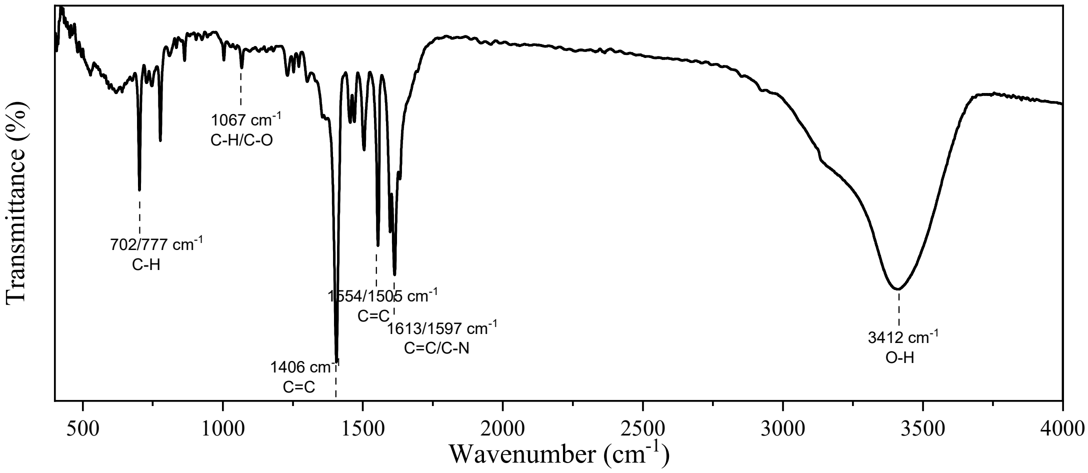

Figure S12: FTIR spectrum of **Ir1**

Table S1. FTIR spectral analysis of **Ir1**

**Band (cm^{-1})**	**Stretching Vibration Assignment**	**Reference**
3412	-OH	[3]
1613	C=C	[4-6]
1597	C=C/C-N	[4,5]
1505	C=C	[7-9]
1406	C=C	[4,5]

1067	C-H/C-O	[3,9,10]
1005	C-H	[3,9,10]
777	C-H	[3,9]
702	C-H	[3,9]

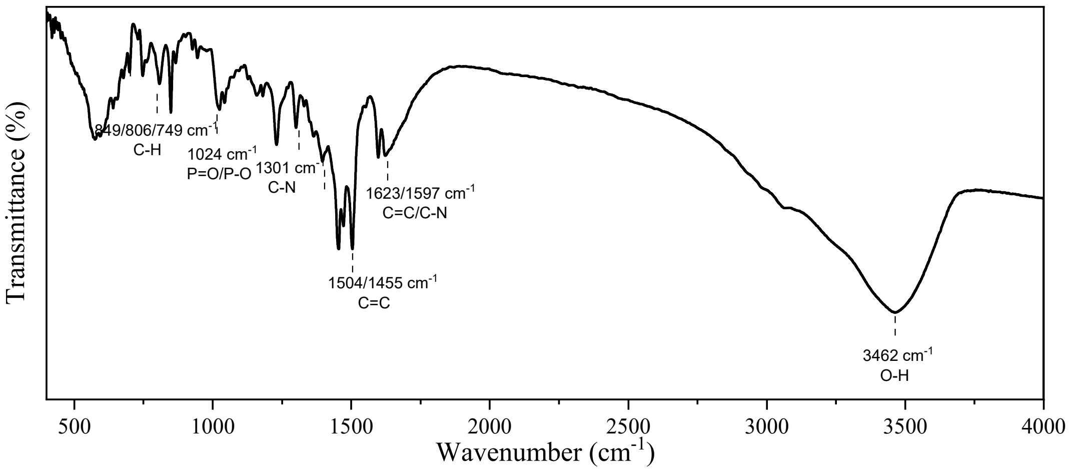

Figure S13: FTIR spectrum of **Ir2**

Table S2. FTIR spectral analysis of **Ir2**

**Band (cm^{-1})**	**Stretching Vibration Assignment**	**Reference**
3462	-OH	[3]
1623	C=C	[4-6]
1597	C=C/C-N	[4,5]
1504	C=C	[7-9]
1455	C=C	[9]
1301	C-N	[3,5]
1024	P-O/P=O	[11]
849	C-H	[3,9,10]
806	C-H	[3,9]
749	C-H	[3,9]

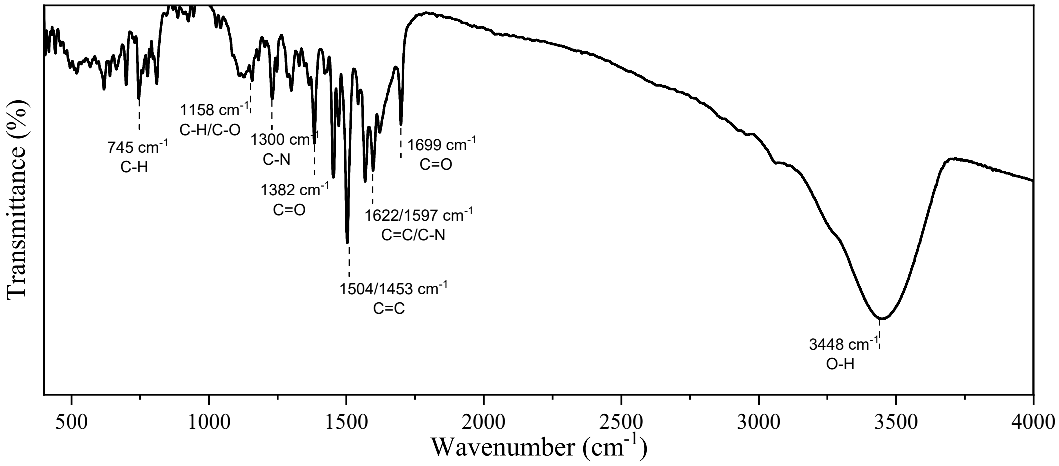

Figure S14: FTIR spectrum of **Ir3**

Table S3. FTIR spectral analysis of **Ir3**

**Band (cm^{-1})**	**Stretching Vibration Assignment**	**Reference**
3448	-OH	[3]
1699	C=O	[12]
1622	C=C	[4-6]
1597	C=C/C-N	[4,5]
1504	C=C	[7-9]
1453	C=C	[9]
1382	C=O	[12]
1300	C-N	[3,5]
1158	C-H/C-O	[3,9,10,12]
745	C-H	[3,9]

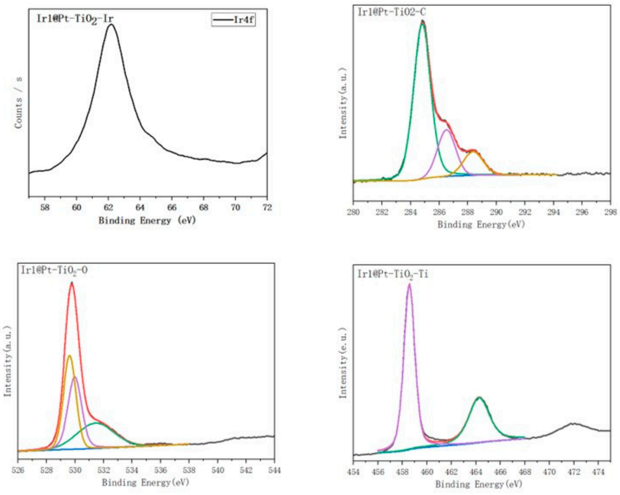

Figure S15: XPS results of **Ir1@Pt-TiO$_2$**

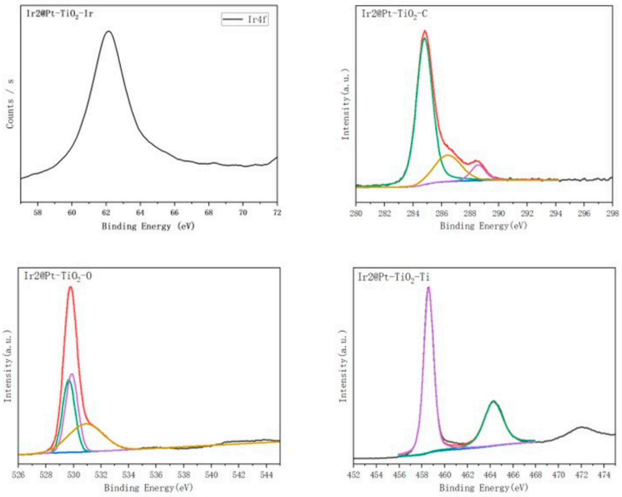

Figure S16: XPS results of **Ir2@Pt-TiO$_2$**

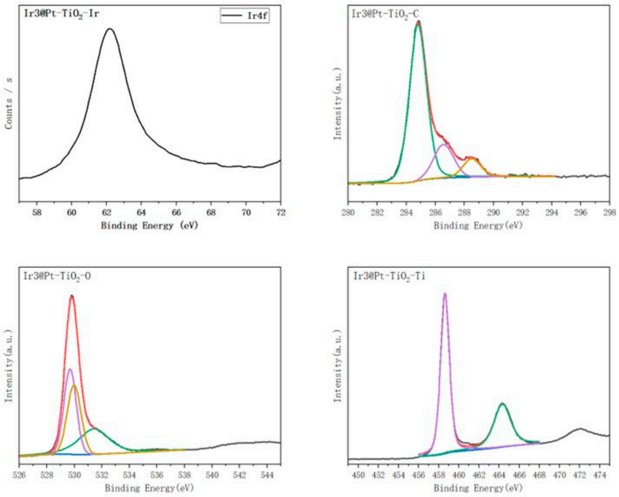

Figure S17: XPS results of **Ir3@Pt-TiO$_2$**

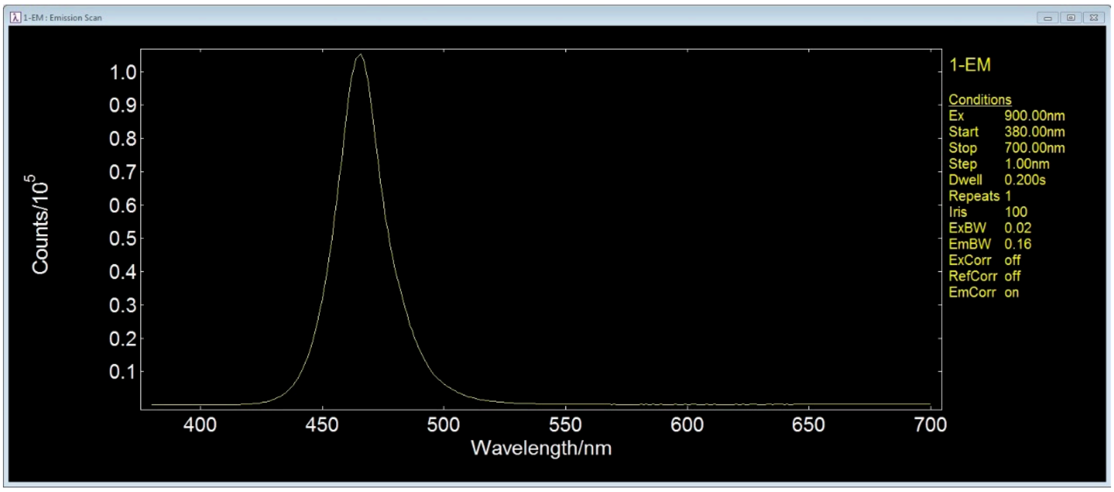

Figure S18: The emission spectra of used LEDs

1. Baglio, J.A.; Calabrese, G.S.; Harrison, D.J.; Kamieniecki, E.; Ricco, A.J.; Wrighton, M.S.; Zoski, G.D. Electrochemical characterization of p-type semiconducting tungsten disulfide photocathodes: efficient photoreduction processes at semiconductor/liquid electrolyte interfaces. *J. Am. Chem. Soc.* **1983**, *105*, 2246–2256.
2. Cho, J.-C.; Park, K.-J.; Ihm, H.-S.; Park, J.-E.; Kim, S.-Y.; Kang, I.; Lee, K.-H.; Jahng, D.; Lee, D.-H.; Kim, S.-J. A novel continuous toxicity test system using a luminously modified freshwater bacterium. *Biosens. Bioelectron.* **2004**, *20*, 338-344.
3. Dahule, H.; Dhoble, S.; Ahn, J.-S.; Pode, R. Synthesis and photophysics of a new deep red soluble phosphorescent iridium (III) complex based on chlorine-methyl-substituted 2, 4 diphenyl quinoline. *J. Phys. Chem. Solids* **2011**, *72*, 1524-1528.
4. Wang, Y.; Zhang, W.; Wu, X.; Luo, C.; Wang, Q.; Li, J.; Hu, L. Conducting polymer coated metal-organic framework nanoparticles: facile synthesis and enhanced electromagnetic absorption properties. *Synth. Met.* **2017**, *228*, 18-24.
5. Liu, P.; Huang, Y.; Zhang, X. Cubic NiFe2O4 particles on graphene–polyaniline and their enhanced microwave absorption properties. *Compos. Sci. Technol.* **2015**, *107*, 54-60.
6. Ge, W.J.; Shao, Q.; Xu, H.; Wan, Y.L. Low Temperature Oxidation Effects on Lignite Molecular Structure. *Adv. Mater. Res.* **2012**, *550*, 2797-2800.
7. Alizadeh, N.; Salimi, A.; Hallaj, R.; Fathi, F.; Soleimani, F. Ni-hemin metal–organic framework with highly efficient peroxidase catalytic activity: toward colorimetric cancer cell detection and targeted therapeutics. *J. Nanobiotechnol.* **2018**, *16*, 1-14.
8. Sun, Z.Y.; Cao, G.P.; Lv, H.; Zhao, L.; Liu, T.; Montastruc, L.; Iordan, N. Equilibrium of benzidine inclusion adsorption on cyclodextrin copolymer. *J. Appl. Polym. Sci.* **2009**, *114*, 3882-3888.
9. Ehrendorfer, C.; Neugebauer, H.; Neckel, A.; Bäuerle, P. An FTIR spectroscopic study on end-capped oligothiophenes as model compounds for polythiophene. *Synth. Met.* **1993**, *55*, 493-498.
10. Hafeez, M.; Afyaz, S.; Khalid, A.; Ahmad, P.; Khandaker, M.U.; Sahibzada, M.U.K.; Ahmad, I.; Khan, J.; Alhumaydhi, F.A.; Emran, T.B. Synthesis of cobalt and sulphur doped titanium dioxide photocatalysts for environmental applications. *J. King Saud Univ. Sci.* **2022**, *34*, 102028.
11. Shih, P.; Yung, S.; Chin, T. FTIR and XPS studies of P_2O_5–Na_2O–CuO glasses. *J. Non-Cryst. Solids* **1999**, *244*, 211-222.
12. Duan, J.; Sontarp, E.J.; Myneni, S.C. Detecting Structural Environments of Carboxyl Groups in Dissolved Natural Organic Molecules. *ACS ES&T Water* **2024**, *4*, 555-563.
